# Supplementary material for: Nigrostriatal dopaminergic neurotransmission and resilience to peripheral systemic risk factors for gait slowing upon transition to uneven surfaces in older adult
Source: Aging Brain. 2025 Jun 20;8:100143. doi: 10.1016/j.nbas.2025.100143 (PMC12221426; doi:10.1016/j.nbas.2025.100143)
Supplement: Supplementary Data 1 [file mmc1.docx]

Supplementary Table 1. Comparison of characteristics of subsamples from each parent cohort

| Variable^i^ |  | MYHAT | | SOMMA | p-value^ii^ | | |
| --- | --- | --- | --- | --- | --- | --- | --- |
|  |  | N=81 | | N=116 |  | | |
| Age (years) |  | 72.51 (4.50) | | 76.60 (3.73) | <0.001 | | |
| Sex |  |  | |  | 0.25 | | |
| Male |  | 27 (33.33) | | 48 (41.38) |  | | |
| Female |  | 54 (66.67) | | 68 (58.62) |  | | |
| Self-Identified Race |  |  | |  | 0.88 | | |
| White |  | 74 (91.36) | | 105 (90.52) |  | | |
| Black |  | 6 (7.41) | | 9 (7.76) |  | | |
| Asian |  | 0 (0.00) | | 1 (0.86) |  | | |
| Native American/Alaskan Native |  | 1 (1.23) | | 0 (0.00) |  | | |
| Unknown |  | 0 (0.00) | | 1 (0.86) |  | | |
| Education |  |  | |  | <0.001 | | |
| Highschool/General Education Development |  | 25 (30.86) | | 7 (6.03) |  | | |
| More than Highschool |  | 56 (69.14) | | 108 (93.10) |  | | |
| Other |  | 0 (0.00) | | 1 (0.86) |  | | |
| Smoking status |  |  | |  | 0.034 | | |
| Never |  | 38 (46.91) | | 67 (57.76) |  | | |
| Past |  | 34 (41.98) | | 46 (39.66) |  | | |
| Current |  | 9 (11.11) | | 3 (2.59) |  | | |
| Body mass index (kg/m^2) |  | 30.91 (5.97) | | 26.85 (4.37) | <0.001 | | |
| Alcohol Intake Past Year |  |  | |  | 0.40 | | |
| No |  | 17 (21.25) | | 31 (26.96) |  | | |
| Yes |  | 63 (78.75) | | 84 (73.04) |  | | |
| Physical Activity: Hours Walked/Week |  | 0.00 (0.00-2.00) | | 1.75 (0.00-3.75) | <0.001 | | |
| Depression Symptoms |  | 0.00 (0.00-1.00) | | 0.00 (0.00-1.00) | 0.32 | | |
| Diabetes |  |  | |  | 0.011 | | |
| No |  | 59 (73.75) | | 102 (87.93) |  | | |
| Yes |  | 21 (26.25) | | 14 (12.07) |  | | |
| Systolic Blood Pressure |  | 133.04 (17.16) | | 130.66 (15.55) | 0.37 | | |
| Diastolic Blood Pressure |  | 75.17 (8.41) | | 70.20 (8.59) | <0.001 | | |
| Pulse |  | 64.15 (8.63) | | 66.46 (10.24) | 0.16 | | |
| Medication Count |  | 3.00 (2.00-5.00) | | 4.00 (2.00-7.00) | 0.29 | | |
| Treated with antidepressant |  | 18 (22.22) | | 19 (16.38) | 0.30 | | |
| Number of comorbidities |  |  | |  | 0.28 | | |
| 0 |  | 37 (46.25) | | 60 (51.72) |  | | |
| 1 |  | 31 (38.75) | | 47 (40.52) |  | | |
| 2 or 3 |  | 12 (15.00) | | 9 (7.76) |  | | |
| Positive Romberg sign^ii^ |  |  | |  |  | | |
| No |  | 78 (100.00) | | 116 (100.00) |  | | |
| Yes |  |  | |  |  | | |
| Vibratory sense reduced in toes^ii^ |  |  | |  | 0.37 | | |
| No |  | 34 (43.59) | | 43 (37.07) |  | | |
| Yes |  | 44 (56.41) | | 73 (62.93) |  | | |
| Pinprick sensation reduced in toes^ii^ |  |  | |  | 0.16 | | |
| No |  | 75 (94.94) | | 114 (99.13) |  | | |
| Yes |  | 4 (5.06) | | 1 (0.87) |  | | |
| Light touch sensation reduced^ii^ |  |  | |  | 0.065 | | |
| No |  | 76 (96.20) | | 116 (100.00) |  | | |
| Yes |  | 3 (3.80) | | 0 (0.00) |  | | |
| White matter hyperintensities (Normalized, mm^3^iii^) |  | 0.006 (0.003-0.009) | | 0.008 (0.004-0.018) | 0.027 | | |
| Low Grip Strength^ii^ |  |  | |  | 0.96 | | |
| No |  | 54 (70.13) | | 81 (69.83) |  | | |
| Yes |  | 23 (29.87) | | 35 (30.17) |  | | |
| Obese |  |  | |  | <0.001 | | |
| No |  | 40 (49.38) | | 88 (75.86) |  | | |
| Yes |  | 41 (50.62) | | 28 (24.14) |  | | |
| Pain for ≥1 month in hip or leg^ii^ |  |  | |  | 0.60 | | |
| No |  | 58 (71.60) | | 79 (68.10) |  | | |
| Yes |  | 23 (28.40) | | 37 (31.90) |  | | |
| Number of peripheral systemic risk factors for gait impairment^iii^ |  |  | |  | 0.22 | | |
| 0 |  | 24 (29.63) | | 43 (37.07) |  | | |
| 1 |  | 29 (35.80) | | 48 (41.38) |  | | |
| 2 |  | 26 (32.10) | | 23 (19.83) |  | | |
| 3 |  | 2 (2.47) | | 2 (1.72) |  | | |
| Gait speed measures (m/s) |  |  | |  |  | | |
| Usual Walking Speed Even Surface |  | | 1.06 (0.18) | 1.12 (0.21) | | 0.05 |  |
| Usual Walking Speed Uneven Surface |  | | 1.00 (0.19) | 1.06 (0.22) | | 0.04 |  |
| Gait speed cost (%GSC) |  | | -5.78 (-9.63--3.00) | -5.14 (-8.55--2.99) | | 0.520 |  |
| DTBZ Binding Potential (BP_ND_) |  | |  |  | |  |  |
| Posterior putamen (sensorimotor striatum) |  | | 2.44 (0.40) | 2.43 (0.43) | | 0.85 |  |
| Anteroventral striatum (limbic striatum) |  | | 1.63 (0.23) | 1.63 (0.29) | | 1.00 |  |
| Caudate and anterior putamen (associative striatum) |  | | 2.08 (0.31) | 2.08 (0.35) | | 0.94 |  |

^i^values shown are N (%), mean (SD), or median (interquartile range)

^ii^p-value for statistical tests comparing MMH to SOMMA with two-sample t-test, two-sample t-test with unequal variance, Wilcoxon rank sum test, chi-square test, or Fisher’s exact test as appropriate.

^iii^Missing values: Romberg n=18, alcohol intake n=2, physical activity n=35, diabetes assessment n=1, BP measurement n=27, WHITE MATTER HYPERINTENSITIES n=3, grip strength n=4, prescription count n=1, sensory exam n=19, joint pain n=1.

^iv^All 3 impairments assessed in n=194; 2 impairments assessed in n=5. In 3 participants no impairments were present but not all 3 impairments were assessed

DTBZ BP_ND_ =dihydrotetrabenazine binding potential, GED=general education diploma . WHITE MATTER HYPERINTENSITIES=white matter hyperintensity

Supplementary Table 2. Relationship between usual gait speed on even and uneven surface and DTBZ binding in those with vs without any peripheral systemic risk factor for gait slowing, adjusted for age and sex

|  | Total sample | | | p-value for  interaction term^ii^ |
| --- | --- | --- | --- | --- |
|  | sβ | SE | p-value^i^ |  |
| Outcome: usual gait speed on even surface |  |  |  |  |
| Predictor: [^11^C]DTBZ BP_ND_ in striatal region |  |  |  |  |
| Posterior putamen (sensorimotor striatum) | 0.116 | 0.073 | 0.11 | 0.44 |
| Anteroventral striatum (limbic striatum) | 0.016 | 0.076 | 0.84 | 0.81 |
| Caudate and anterior putamen (associative striatum) | 0.033 | 0.073 | 0.65 | 0.72 |
| Outcome: usual gait speed on uneven surface |  |  |  |  |
| Predictor: [^11^C]DTBZ BP_ND_ in striatal region |  |  |  |  |
| Posterior putamen (sensorimotor striatum) | 0.026 | 0.015 | 0.09 | 0.89 |
| Anteroventral striatum (limbic striatum) | 0.004 | 0.016 | 0.82 | 0.62 |
| Caudate and anterior putamen (associative striatum) | 0.011 | 0.015 | 0.48 | 0.89 |

^i^ Linear regression model consisting of: gait speed cost on transition from even to uneven surface (outcome), [^11^C]DTBZ BP_ND_ in specified striatal region, age, and sex

^ii^ Linear regression model consisting of: gait speed cost on transition from even to uneven surface (outcome), [^11^C]DTBZ BP_ND_ in specified striatal region, age, and sex, presence of any risk factor, and interaction term between risk factor present (yes/no) and [^11^C]DTBZ BP_ND_ in specified striatal region

DTBZ BP_ND_ =dihydrotetrabenazine binding potential. PSRF=peripheral systemic risk factors. sβ=standardized beta coefficient. SE=standard error
